# The first fossil salmonfly (Insecta: Plecoptera: Pteronarcyidae), back to the Middle Jurassic

**DOI:** 10.1186/s12862-016-0787-9

**Published:** 2016-10-18

**Authors:** Yingying Cui, Olivier Béthoux, Boris Kondratieff, Chungkun Shih, Dong Ren

**Affiliations:** 1 Sorbonne Universités, UPMC Univ Paris 06, MNHN, CNRS, Centre de recherche sur la paléobiodiversité et les paléoenvironnements (CR2P), Paris, France; 2 College of Life Sciences, Capital Normal University, Beijing, 100048 China; 3 Department of Bioagricultural Sciences and Pest Management, Colorado State University, Fort Collins, CO 80523 USA; 4 Department of Paleobiology, National Museum of Natural History, Smithsonian Institution, Washington, DC 20013-7012 USA

## Abstract

**Background:**

The fossil record of Plecoptera (stoneflies) is considered relatively complete, with stem-groups of each of the three major lineages, viz. Antarctoperlaria, Euholognatha and Systellognatha (and some of their families) represented in the Mesozoic. However, the family Pteronarcyidae (the salmonflies; including two genera, *Pteronarcys* and *Pteronarcella*) has no fossil record to date, and the family has been suggested to have diverged recently.

**Results:**

In this paper, we report on a set of specimens belonging to a new fossil species of stonefly, discovered from the Middle Jurassic Daohugou locality (China). Our comparative analysis of wing venation and body characters demonstrates that the new species belongs to the Pteronarcyidae, and is more closely related to *Pteronarcys* than to *Pteronarcella*. However, it differs from all known species of the former genus. It is therefore assigned to a new genus and named *Pteroliriope sinitshenkovae* **gen. et sp. nov.** under the traditional nomenclatural procedure. The cladotypic nomenclatural procedure is also employed, with the resulting combination *Pteroliriope nec Pteronarcys sinitshenkovae* **sp. nov.**

**Conclusions:**

The first discovery of a fossil member of the Pteronarcyidae demonstrates that the corresponding lineage is not a very recent offshoot but was already present ca. 165 million years ago. This discovery concurs with the view that divergence of most stonefly families took place very early, probably in the Triassic, or even in the Permian. This contribution demonstrates the need for (re-)investigations of the systematics of fossil stoneflies to refine divergence date estimates for Plecoptera lineages.

## Background

The insect order Plecoptera (stoneflies) diverged as early as in the early Late Carboniferous [1], and stem-relatives of most of its constituent families have been recovered from Mesozoic strata [2–9]. However the Pteronarcyidae (salmonflies; including two extant genera, *Pteronarcys* Newman, 1838 [10] and *Pteronarcella* Banks, 1900 [11]), a prominent family as far as the size of individuals is concerned, has never been recovered in the fossil record. As a consequence it is generally assumed that the family diverged very recently (Cenozoic [2, 4]).

In this paper, we report on a large set of specimens belonging to a new fossil species of stonefly, unearthed from the Middle Jurassic Daohugou locality (China). Our comparative analysis of wing venation and body characters unambiguously demonstrates that the new species is a Pteronarcyidae. Its affinities within this family are elucidated and the implications of this new occurrence on divergence date estimates for stonefly lineages are addressed.

## Results

In order to assess the affinities of a newly discovered fossil species, we conducted a comparative analysis of characters of the external morphology. Firstly we complemented the available data on the wing venation of *Pteronarcella badia* (Hagen 1874) [12], a close relative of the new species. Then we address systematics aspects above the species level (divided into the traditional and the cladotypic approaches), followed by species-level aspects.

### Wing venation variability in *Pteronarcella badia*

**Fig. 1:**
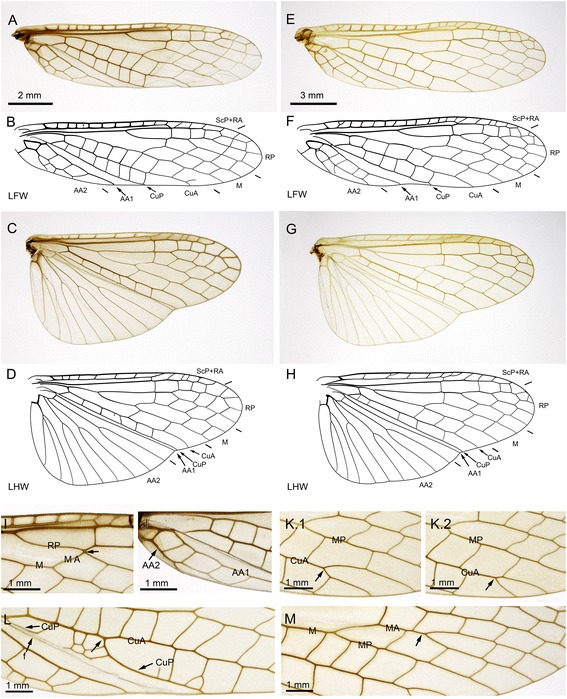
Wing venation of *Pteronarcella badia* (Hagen, 1874). **a-d**, Specimen MNHN.EP654 (*male*), photographs and drawings of left forewing and left hind wing; (**e-h**), Specimen MNHN.EP657 (*female*), photographs and drawings of left forewing and left hind wing; (**i-m**), photos of detail of forewing venation variability; (**i**), Specimen MNHN.EP655 (*male*), left forewing; (**j**), Specimen MNHN.EP656 (*male*), left forewing; (**k**).1-K.2, Specimen MNHN.EP658 (*female*), right and left forewing respectively; (**l**), Specimen MNHN.EP661 (*female*), left forewing; (**m**), Specimen MNHN.EP662 (*female*), right forewing

During our survey, we discovered that there was a lack of data on the wing venation of *Pteronarcella badia*, a critical species for comparison. An incomplete view of a forewing is available in Needham & Claassen (1925) [13] (Fig. 11), and a fore- and a hind wing are illustrated by Nelson & Hanson 1971 [14]: (Figs. 23–26). We investigated nine macropterous specimens (three males, six females). The typical morphology of the species is represented by Fig. 1a–d for males and Fig. 1e–h for females. In both fore- and hind wing, it involves a 3- or 4-branched RP, a 2-branched M (i.e. both MA and MP simple), a simple CuA, and no cross-veins in the area between R/RP and M basal to the first fork of M. In forewings, the rp-ma cross-vein is long, and AA2 has 2–3 branches.

Unusual morphologies were documented: one individual exhibited a short rp-ma cross-vein in both forewings (Fig. 1i.1,2); one individual exhibited a CuA forked distally in both forewings (only left forewing represented on Fig. 1i); one individual exhibited a long stem of AA2 (Fig. 1j); another one, not illustrated, has also such a distal fork in one of the forewings (Fig. 1k.1,2); one individual has CuP fused for some distance with CuA (Fig. 1l); one individual has a branched MA (Fig. 1m); one unusual individual has an incomplete cross-vein in the area between R/RP and M basal to the fork of M in the right forewing, and MP fused with CuA in the right hind wing, among other aspects (not illustrated).

### Systematics above species level

#### Traditional nomenclature

**Plecoptera** Burmeister, 1839

**Systellognatha** Zwick, 2000

**Pteronarcyidae** Newman, 1853

***Pteroliriope*** Cui, Béthoux, Kondratieff, Shih & Ren, **gen. nov.**

(urn:lsid:zoobank.org:act:514D4664-70D2-4A89-AFA1-B9B9423052D8)

**Fig. 2:**
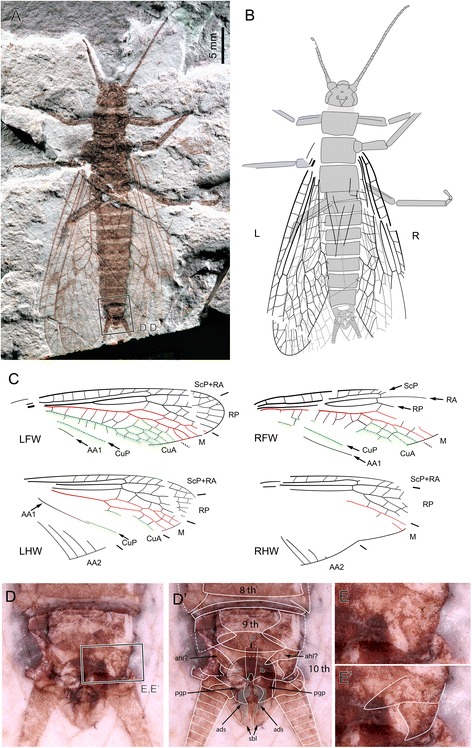
*Pteroliriope sinitshenkovae* tax. et sp. nov. (Middle Jurassic; Daohugou, China), specimen CNU-PLE-NN-2015001, holotype; (**a**), Photograph (composition of positive and negative imprints); (**b-c**), Drawings; (**d-d’**), Detail photograph (composition of positive and negative imprints, both dry and under ethanol, reversed) of posterior part of male postabdomen, without (**d**) and with (**d’**) interpretation, as located on (**a**); (**e-e’**), Detail photograph (composition of positive and negative imprints, both dry and under ethanol, reversed) of hemitergal lobe, without (**e**) and with (**e’**) interpretation, as located on (**d**)

**Fig. 3:**
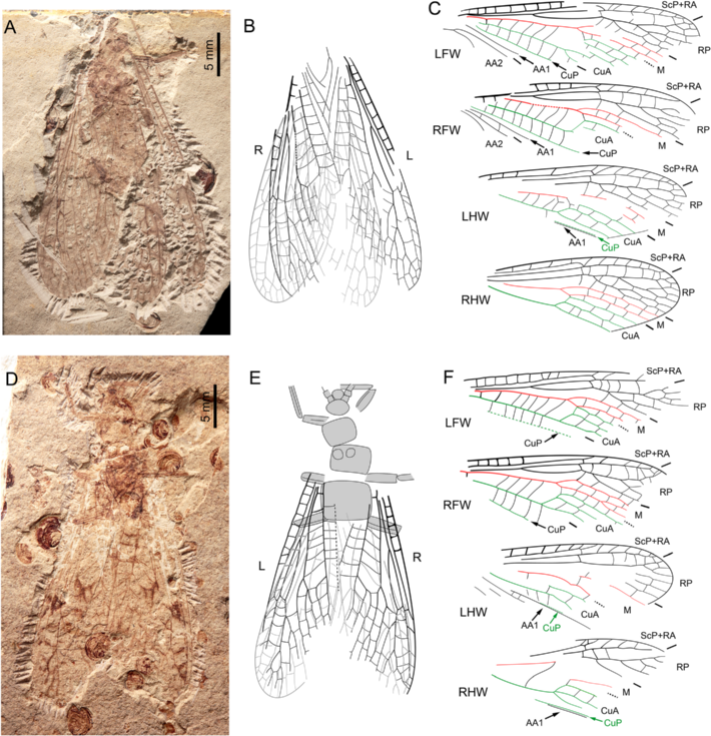
*Pteroliriope sinitshenkovae* tax. et sp. nov. (Middle Jurassic; Daohugou, China). **a-c**, Specimen CNU-PLE-NN-2013001; (**a**), Photograph (negative imprint); (**b-c**), Drawings; (**d-f**), Specimen CNU-PLE-NN-2013004; (**d**), Photograph (positive imprint); (**e-f**), Drawings

**Fig. 4:**
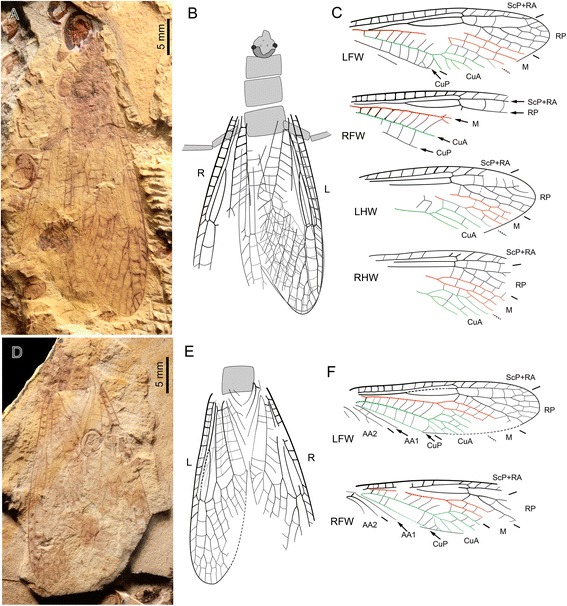
*Pteroliriope sinitshenkovae* tax. et sp. nov. (Middle Jurassic; Daohugou, China). **a-c**, Specimen CNU-PLE-NN-2013003; (**a**), Photograph (negative imprint); (**b-c**), Drawings; (**d-f**), Specimen CNU-PLE-NN-2013002; (**d**), Photograph (positive imprint); (**e-f**), Drawings

**Fig. 5:**
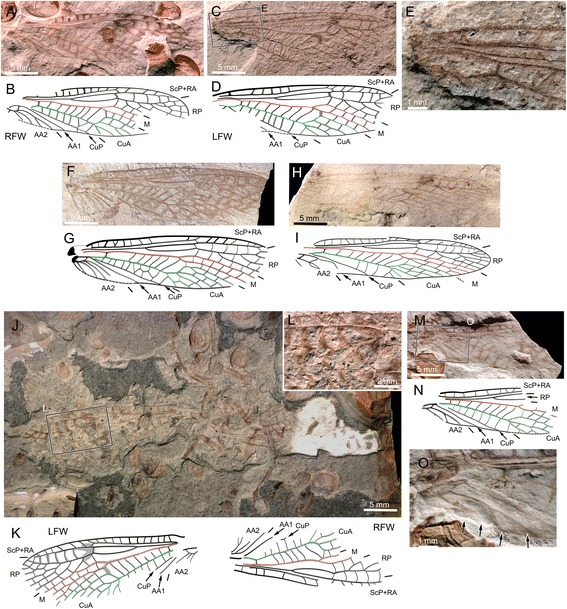
*Pteroliriope sinitshenkovae* tax. et sp. nov. (Middle Jurassic; Daohugou, China). **a-b**, Specimen CNU-PLE-NN-2013024; (**a**), Photograph (positive imprint, right forewing); (**b**), Drawing; (**c-e**), Specimen CNU-PLE-NN-2013021; (**c**), Photograph (negative imprint); (**d**), Drawing; (**e**), Detail of anterior-basal area (as located on **c**), the arrow indicates the ‘systellognathan’ basal oblique cross-vein; (**f-g**), Specimen CNU-PLE-NN-2013020; (**f**), Photograph (positive imprint); (**g**), Drawing; (**h-i**), Specimen CNU-PLE-NN-2013006; (**h**), Photograph (positive imprint); (**i**), Drawing; (**j-l**), Specimen CNU-PLE-NN-2013032; (**j-k**), Photograph (positive imprint of left forewing and negative imprint of right forewing) and drawings; (**l**), Detail photo of coloration in LFW, as located on (**j**); (**m-o**), Specimen CNU-PLE-NN-2013019; (**m-n**), Photograph (positive imprint, reversed) and drawing; (**o**), Detail of basal part of forewing, arrows showing branches of AA2, as located on (**m**)

**Fig. 6:**
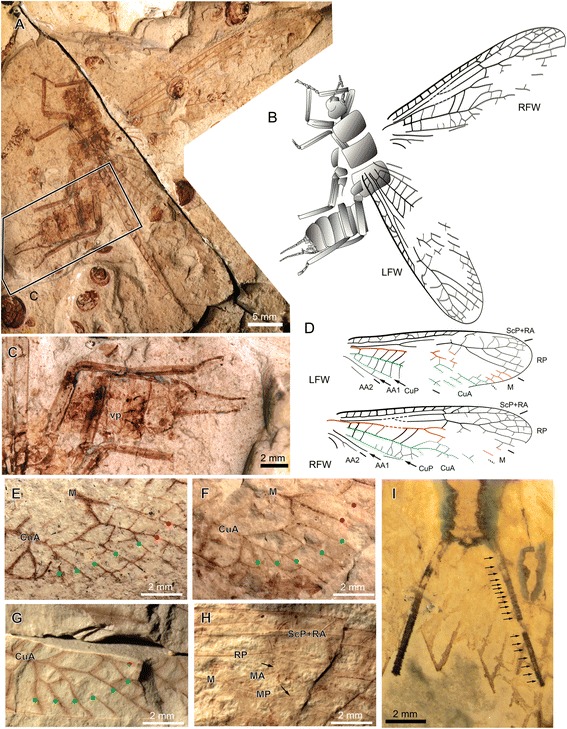
*Pteroliriope sinitshenkovae* tax. et sp. nov. (Middle Jurassic; Daohugou, China). **a-d**, Specimen CNU-PLE-NN-2013005; (**a**), Photograph (positive imprint); (**b,d**), Drawings; (**c**), Detail of the end of abdomen and legs, as located on (**a**); (**e-h**), Photographs of detail forewing venation variability, branches of M are indicated by red spots, branches of CuA are indicated by green spots; (**e**), Specimen CNU-PLE-NN-2013007; (**f**), Specimen CNU-PLE-NN-2013008; (**g**), Specimen CNU-PLE-NN-2013027; (**h**), Specimen CNU-PLE-NN-2013026; (**i**), Detail of cerci of specimen CNU-PLE-NN-2013011

**Fig. 7:**
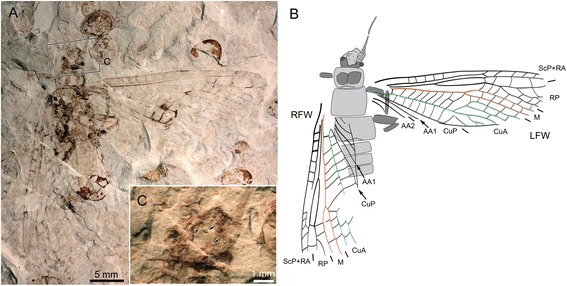
*Pteroliriope sinitshenkovae* tax. et sp. nov. (Middle Jurassic; Daohugou, China), specimen CNU-PLE-NN-2013036; (**a**), Photograph (negative imprint); (**b**), Drawing; (**c**), Detail of head, arrows showing three ocelli, as located on (**a**)

**Type species:** *Pteroliriope sinitshenkovae* **gen. et sp. nov.** (Figs. 2, 3, 4, 5, 6, 7 and 9A.2)

**Diagnosis:** By monotypy, that of its constituent species.

**Etymology:** Based on ‘Pteron’ (‘wing’ in ancient Greek), and ‘Liriope’, a nymph considered the mother of Narcissus in Greek mythology (the name of a related genus, *Pteronarcys*, is based on ‘Narcissus’).

**Composition:** *Pteroliriope sinitshenkovae* **gen. et sp. nov.**

**Discussion:** The type-species of the proposed new genus exhibits a unique combination of character states (see below, main Discussion), justifying the described new genus.

#### Cladotypic nomenclature

Taxon ***Pteroliriope*** Cui, Béthoux, Kondratieff, Shih & Ren, **tax. nov.**

**Definition:** Species that evolved from the hypothetical ancestral species in which the character state ‘in forewing, AA2 with more than three branches (as opposed to ‘in forewing, AA2 with three branches, or less’), as exhibited by *californica* Newport, 1851 and *princeps* Banks, 1907, has been acquired (venation designations as herein).

**Etymology:** See above.

**Cladotypes:** Specimen MNHN-EP663 (belonging to *californica* Newport, 1851 [15], male), and specimen MNHN-EP667 (belonging to *princeps* Banks, 1907 [16], male).

**Paracladotypes:** Specimens MNHN-EP664 to -EP666 (belonging to *californica* Newport, 1851 [15], two males, one female), and specimens MNHN-EP668 to -EP670 (belonging to *princeps* Banks, 1907 [16], two males, one female).

**Composition:** All recent species currently assigned to the genus *Pteronarcys* (see [17]) and *sinitshenkovae* **sp. nov.**

**Discussion:** Wings of both cladotypic species have been illustrated in a former publication [1]. Systematic aspects are treated under the traditional heading (see above). As for a possible earlier association of the proposed defining character state alone with a taxon (named, or not), we found none.

Taxon ***Pteronarcys*** nom. Newman, 1838, Cui, Béthoux & Kondratieff **dis.-typ. nov.**

**Definition:** Species that evolved from the hypothetical ancestral species in which the character state ‘in forewing, area between R/RP and M basal to the fork of M broad, with cross-veins’ (as opposed to ‘in forewing, area between R/RP and M basal to the fork of M of regular width, without cross-veins’), as exhibited by *californica* Newport, 1851 and *princeps* Banks, 1907, has been acquired (venation designations as herein).

**Cladotypes:** Specimen MNHN-EP663 (belonging to *californica* Newport, 1851 [15], male), and specimen MNHN-EP667 (belonging to *princeps* Banks, 1907 [16], male).

**Paracladotypes:** Specimens MNHN-EP664 to -EP666 (belonging to *californica* Newport, 1851 [15], two males, one female), and specimens MNHN-EP668 to -EP670 (belonging to *princeps* Banks, 1907 [16], two males, one female).

**Composition:** All recent species currently assigned to the genus *Pteronarcys* (see [17]). The species *sinitshenkovae* **sp. nov.** can be conclusively excluded from this taxon (see below).

**Discussion:** Wings of both cladotypic species have been illustrated in an earlier publication [1]. The character state defining the taxon *Pteronarcys* (name first erected in Newman, 1837 ﻿[10]) can be seen as composed of two characters regarding the area between R/RP and M, and basal to the fork of M, viz. its width (regular vs. broad), and the occurrence of cross-veins (absent vs. present). However, to our knowledge, the state ‘area of regular width’ always co-occurs with the state ‘cross-veins absent’, and the state ‘area broad’ always co-occurs with the state ‘cross-veins present’. Indeed a broadening of the space between two veins is likely to decrease the repression of cross-veins formation. In other words, the two conditions are most likely correlated.

Our literature survey revealed no earlier association of the proposed defining character state along with a taxon (named or not, we found no mention of the defining character state). Because the composition of the taxon herein defined matches that of the genus *Pteronarcys*, we propose to adapt this name under cladotypic nomenclature.

### Species-level systematics

***Pteroliriope sinitshenkovae*** **Cui, Shih, & Ren, sp. nov.** (Figs. 2, 3, 4, 5, 6, 7 and 9A.2)

(urn:lsid:zoobank.org:act:94D0E24A-C812-4C83-8951-BB4DA7661EFA)

**Diagnosis:** *Forewing:* area between R/RP and M basal to the fork of M of regular width, without cross-veins (shared with *Pteronarcella* spp.; opposite condition in *Pteronarcys* spp.); MA distinct from RP (shared with *Pteronarcella* spp., fusion occasionally present in *Pteronarcys* spp.); AA2 with more than 3 branches (shared with *Pteronarcys* spp.; less than 3 branches in *Pteronarcella* spp.); except for the aa_1_-aa_2_ cross-vein, no cross-vein between AA2 branches (cross-veins present in both *Pteronarcella* spp. and *Pteronarcys* spp.). *Hind wing:* CuA branched (simple in *Pteronarcella* spp., branched in *Pteronarcys* spp.).

**Etymology:** The specific eptithet honours Dr. N. Sinitshenkova, for her important contributions to the study of fossil stoneflies.

**Material:** Holotype specimen: CNU-PLE-NN-2015001; paratype specimens: CNU-PLE-NN-2013001, CNU-PLE-NN-2013005; other specimens: CNU-PLE-NN-2013002 – CNU-PLE-NN-2013004, CNU-PLE-NN-2013006 – CNU-PLE-NN-2013008, CNU-PLE-NN-2013011, CNU-PLE-NN-2013019 – CNU-PLE-NN-2013021, CNU-PLE-NN-2013024, CNU-PLE-NN-2013026, CNU-PLE-NN-2013027, CNU-PLE-NN-2013032, CNU-PLE-NN-2013036.

**Locality:** near Daohugou Village, Ningcheng County, Inner Mongolia, China; Jiulongshan Formation; late Middle Jurassic [18–20].

**General description:** *Body*: total length (excluding antennae and cerci) ca. 25 mm; head narrower than prothorax, with basal part broader than distal part; antennae filiform; three ocelli; eyes round, dark-coloured; prothorax, mesothorax and metathorax of similar rectangular shape, width about 1.5 times long as length; forelegs shortest, hind legs longest; femur robust; tibia slender; tarsus with three segments, second one shortest, third one (pretarsus) longest; pretarsus with a pair of simple claws; cerci multi-segmented. *Forewings:* average length 28.1 mm (longest 31.4 mm in specimen CNU-PLE-NN-2013011, shortest 24.1 mm in specimen CNU-PLE-NN-2013035), average width 7.5 mm (widest 8.8 mm in specimen CNU-PLE-NN-2013029, narrowest (5.7 mm) in specimen CNU-PLE-NN-2013015) (dimensions based on 18 specimens preserving almost-complete forewings); occurrence of a strong oblique cross-vein present in area between the anterior wing margin and ScP close to wing base (Fig. 5c–e); more distally, area between anterior margin and ScP filled with strong cross-veins; ScP reaches RA at second third of wing length; several cross-veins (branches from ScP + RA?) occurring between ScP + RA and anterior margin; RA-RP fork in second fifth of wing length; area between RA and RP with a constriction opposite ra-rp cross-vein; RP with 4–8 branches (including distalmost one or two); in area between RA and RP, first cross-vein slightly basal to the end of ScP on RA, stronger than other following cross-veins; M bent downward slightly before its fork; fork located near wing mid-length; MA mostly simple; MP simple or forked; a strong cross-vein (arculus) between base of M and CuA; CuA forked distally, slightly basal to the fork of M; CuA with 4(3?)-8 branches; CuP straight, simple; AA1 simple; AA2 normally with 4 branches, with varying branching pattern; dark pigmentation along most of venation, especially obvious along ra-rp cross-vein, cross-veins in area between anterior wing margin and ScP, and RP at its fork. *Hind wings:* visible parts very similar to forewings except: area between anterior wing margin and ScP + RA, slightly wider; and CuA with 3 (2?) branches.

**Specimen descriptions:** Specimen CNU-PLE-NN-2015001 (holotype; Fig. 2): complete individual, male, positive and negative imprints, wings partly overlapping. *Body:* posterior margin of head 4.5 mm long; outline of mouthpart visible; antennae with 28 segments preserved; prothorax 3.0 mm long, 4.9 mm wide; mesothorax 3.5 mm long, 5.0 mm wide; metathorax 3.5 mm long, 5.5 mm wide; femur of foreleg about 4.0 mm long, 1.0 mm wide; tibia not completely preserved, 0.6 mm wide; femur of right mid-leg 3.5 mm long, 1.7 mm wide; tibia of left mid-leg 7.0 mm long, 0.5 mm wide; femur of left hind leg about 5.0 mm long, 1.0 mm wide; tibia of right hind leg 7.0 mm long, 0.7 mm wide; tarsus 3-segmented; first segment 1.0 mm long, second segment 0.5 mm long, third segment (pretarsus) 1.1 mm long; abdomen completely preserved; ninth sternite, posterior edge of tergum with two hook-like structures consisting of three lobes; anterior hemitergal lobe (ahl) comparatively long and narrow, well separated from median lobe (mhl); mhl and posterior (phl) hemitergal lobes processes; tenth tergum divided mid-dorsally into two hemitergites; each hemitergite not clearly separated from each other, with posterior lobe small and pointed; inner part (ip) of supra-anal process visible; two lateral braces (lb) at the apex of ip, very broad; paragenital plate (pgp) with anterior lateral edged terminating close to lateral braces (lb); apical region of dorsal section of supra-anal process (ads) partly visible; eleventh segment divided into two hook-like sub-anal lobes (sbl). *Left forewing*: 30.0 mm long and 7.9 mm wide; base of Cu and most part of anal area not visible. *Right forewing*: 25.4 mm long and 10.0 mm wide as preserved; anterior-distal area not preserved; posterior-basal area not visible except CuP and AA1. *Left hind wing*: 28.5 mm long, 8.9 mm wide as preserved; CuA mostly not visible; AA2 with 4 branches reaching posterior margin visible. *Right hind wing*: 20.0 mm long, 9.0 mm wide as preserved; distal part of wing not preserved; area between anterior branch of M and three posterior branches of AA2 not identifiable.

Specimen CNU-PLE-NN-2013001 (paratype; Fig. 3a–c): four wings overlapping. *Left forewing*: 29.6 mm long and 6.9 mm wide as preserved; base of Cu not visible. *Right forewing*: 27.1 mm long and 7.9 mm wide as preserved, with very basal and posterio-distal part not visible; arculus, branches of CuA, and basal forks of AA2 not visible. *Hind wings:* CuA with 3 branches. *Left hind wing*: 24.0 mm long and 8.5 mm wide as preserved; fork of R and M not visible; distal part of CuP and AA1 visible. *Right hind wing*: 22.1 mm long, 8.5 mm wide as preserved.

Specimen CNU-PLE-NN-2013004 (Fig. 3d–f): Head and thorax preserved with all three pairs of legs; fore- and hind wing overlapping on both sides. *Left forewing*: 24.9 mm long, 7.0 mm wide preserved, with distal part, posterior margin and anal area not visible. *Right forewing*: 23.5 mm long, 7.7 mm wide as preserved; MP forked. *Left hind wing*: basal part and posterior margin not visible; 22.6 mm long and about 8.0 mm wide as preserved. *Right hind wing*: veins partly visible, basal and distal part missing; 19.8 mm long and 8.6 mm wide as preserved.

Specimen CNU-PLE-NN-2013003 (Fig. 4a–c): Head, thorax and hind legs preserved, four wings overlapping, in resting position. *Left forewing*: about 32.0 mm long, and 10.6 mm wide, as preserved; basal part uninterpretable. *Right forewing*: 27.0 mm long and 9.7 mm wide as preserved; mid-anterior part visible. *Left hind wing*: 29.3 mm long, 10.9 mm wide. *Right hind wing*: 24.6 mm long, 11.6 mm wide; MP forked.

Specimen CNU-PLE-NN-2013002 (Fig. 4d–f): a pair of wings in resting position, with a few veins of left hind wing visible. *Left forewing*: nearly complete, 26.4 mm long, 6.9 mm wide; MP forked. *Right forewing*: incompletely preserved, with distal part missing; 22.7 mm long, 7.0 mm wide; as preserved, MP simple.

Specimen CNU-PLE-NN-2013024 (Fig. 5a–b): positive imprint of right forewing; 27.6 mm long and 7.5 mm wide; CuA with 5 branches, all distinct from M.

Specimen CNU-PLE-NN-2013021 (Fig. 5c–e): negative imprint of left forewing; 26.6 mm long and 7.6 mm wide as preserved; CuA with 5 branches, all distinct from M.

Specimen CNU-PLE-NN-2013020 (Fig. 5f–g): positive and negative imprint of right forewing; 30.3 mm long and 7.8 mm wide as preserved; coloration well preserved; CuA with 6 branches, all distinct from M; cross-veins in the area between CuA and CuP reticulated.

Specimen CNU-PLE-NN-2013006 (Fig. 5h–i): positive imprint of a nearly complete right forewing; 29.4 mm long, 6.0 mm wide; MP forked; CuA with 5 branches, one fused with MP.

Specimen CNU-PLE-NN-2013032 (Fig. 5j–l): positive imprint of left forewing and negative imprint of right forewing, with scattered legs. *Left forewing:* 26.5 mm long and 8.6 mm wide as preserved; MP forked; CuA with 8 branches, one fused with MP; *Right forewing:* 23.2 mm long, 8.6 mm wide as preserved.

Specimen CNU-PLE-NN-2013019 (Fig. 5m–o): positive imprint of left forewing; 19.4 mm long and 6.9 mm wide as preserved; CuA with a branch fused with MP; base very well preserved, with 4 distinct branches of AA2.

Specimen CNU-PLE-NN-2013005 (paratype; Fig. 6a–d): nearly complete body and both forewings preserved, female. *Body:* basal part of head 2.3 mm long; antennae with 8 segments preserved; prothorax 3.2 mm long, 4.7 mm wide; mesothorax 2.9 mm long, 4.6 mm wide; metathorax largest, 3.6 mm long, 5.4 mm wide; forelegs well preserved; femur about 4.3 mm long, 1.0 mm wide; tibia about 4.6 mm long, 0.6 mm wide; femur of mid-leg about 3.9 mm long, 0.9 mm wide; tibia of mid-leg 5.0 mm long, 0.6 mm wide; femur of hind leg about 4.9 mm long, 0.9 mm wide; tibia of hind leg 7.0 mm long, 0.7 mm wide; eight sternite with two round vaginal projections (vp, in Fig. 6c). *Left forewing*: 26.9 mm long, 8.0 mm wide; MP probably forked; *Right forewing*: 29.3 mm long, 7.4 mm wide.

Specimen CNU-PLE-NN-2013036 (Fig. 7a–c): half body and both forewings preserved; head 3.5 mm wide between compound eyes; prothorax 3.1 mm long, 4.6 mm wide; mesothorax 3.9 mm long, 5.2 mm wide; metathorax 3.4 mm long, 6.1 mm wide; three abdominal segments visible. *Left forewing*: 26.1 mm long, 8.3 mm wide as preserved; *Right forewing*: preserved 22.4 mm long, 8.2 mm wide.

**Discussion:** The interpretation of the genitalia of specimen CNU-PLE-NN-2015001 is based on a comparison with the description of extant taxa of the Pteronarcyidae by Nelson and Hanson (1971; [14]) on extant Pteronarcyidae, and is included in the main Discussion section.

The distinction between the conditions ‘branched MP’ and ‘fusion of a branch of CuA with MP’ proved difficult to confirm in several cases. For example, in the case of the specimen CNU-PLE-NN-2013002, the left forewing is interpreted to have a branch of CuA fused with MP (Fig. 4f), but a branched MP remains a plausible interpretation, given that the presumed MP fork is located in a very basal position. However, the branching pattern of CuA in the forewing shows variation in the number of branches, usually with four to five branches, rarely with three (Fig. 3f) or six or more (Figs. 4c, 5g, k and 6g).

We found no reason to distinguish different species within the fossils we examined. Data on extant species of *Pteronarcys* ([1]; below considered as closely related to the new fossil species) show that the observed size variation, and the occurrence of a branched MP, is subjected to intra-specific variation. The branching pattern of AA2 in forewing of Pteronarcyidae (with which the new species is closely related, see below) also shows a certain degree of variation, in particular in *Pteronarcys* spp. (see Figs. 23, 24 in [21]), which can also be observed in the fossil specimens: the first branch of AA2 can be simple or forked (Fig. 4f, compare LFW and RFW); and the first branch of AA2α branched distally or proximally (compare Fig. 5b and g). Therefore, the assignment of all the specimens listed above to a single species is well supported.

## Discussion

Before proceeding with the evolutionary implications of our discovery, a note on nomenclatural aspects is necessary. Under the cladotypic nomenclature all taxon names are written in italics with a capital letter. Traditional genus names and cladotypic names can therefore be confused. However, the name of the newly erected genus (viz., ‘*Pteroliriope*’) is also used in association with a cladotypic definition. Consequently the combination ‘*Pteroliriope sinitshenkovae*’ is valid under both procedures. In addition the adaptation of the pre-existing genus name ‘*Pteronarcys*’ under cladotypic nomenclature leaves the association of this name with the specific epithets of its constituent species unchanged (e.g., ‘*Pteronarcys californica*’). In such cases both procedures reach a similar outcome [22]. However the cladotypic procedure has that advantage that the exclusion of *sinitshenkovae* from the taxon *Pteronarcys* can be made explicit with the minimal combination ‘*Pteroliriope nec Pteronarcys sinitshenkovae*’ (this cannot be achieved under the traditional procedure without inflating ranks [23] and a good knowledge of the corresponding group systematics). Unless mentioned, this section will be further continued under the cladotypic framework. In other words, all taxon names written in italics are to be understood as cladotypic names. Species are referred to by their specific epithet associated with authorship data (also known as Lanham species names).

### Systematic affinities of *Pteroliriope sinitshenkovae* tax. et sp. nov.

**Fig. 8:**
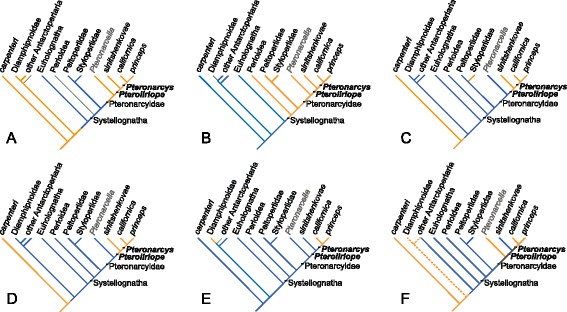
Distribution of selected character states, (phylogeny after [32], complemented by data from [1] and [41]; ‘*Pteroliriope*’ placed as under the cladotypic approach, i.e. it is a taxon which includes the species *sinitshenkovae* sp. nov. and the taxon *Pteronarcys* dis.-typ. nov.). **a**, In the distal half of the wing, in the areas between branches of M and CuA, crossveins absent (*blue*) vs. numerous (*orange*); (**b**), First cross-vein in the area between the anterior wing margin and ScP not short, strong and oblique (*blue*) vs. short, strong and oblique; (**c**), Forewing, number of AA2 branches, three or less (*blue*) vs. more than three (*orange*); (**d**), Fore- and hind wing, M occasionally with more than two branches, no (*blue*) vs. yes (*orange*); (**e**), Forewing, area between R/RP and M basal to the fork of M with regular width and without cross-veins (*blue*) vs. broad and with cross-veins (*orange*); (**f**), Forewing, cross-veins in the areas between branches of AA2, none occurring (*blue*) vs. occurring (*orange*)

The distribution of the states of selected characters is summarized in Fig. 8. At first glance, the state ‘in the distal half of the wing, in the areas between branches of M and CuA, occurrence of numerous cross-veins’ (as opposed to ‘no cross-veins’) is a very distinctive trait (orange on Fig. 8a). This trait is shared with *carpenteri* Béthoux et al., 2011 [1], which is sister-group related to all other Plecoptera, with most Antarctoperlaria (with the exception of some Gripopterygidae), and, within the Systellognatha, with some Perlidae, *zhaoi* Liu et al. 2007 [24] (a fossil Systellognatha of unclear affinities [3]), and the Pteronarcyidae.

The occurrence of a short, oblique strong first cross-vein in the area between the anterior wing margin and ScP (arrow on Fig. 5c–e) allows the assignment of *sinitshenkovae* **sp. nov.** to the Systellognatha, as this state is unique to the group (orange on Fig. 8b). The character state ‘in hind wing, CuA branched’ (as opposed to ‘simple’) is congruent with the previous one: indeed this state occurs only in some Diamphipnoidae and in Systellognatha (with the exception of species of the genus *Pteronarcella*; state distribution not represented on Fig. 8). The assignment of *sinitshenkovae* **tax. et sp. nov.** to the Systellognatha, and, more specifically to the Pteronarcyidae, is well corroborated.

The character state ‘in forewing, AA2 with more than three branches’ (as opposed to ‘with three or fewer branches’; orange on Fig. 8c) confirms this statement: among Plecoptera it is known only in *carpenteri*, the Styloperlidae (see below), and *Pteronarcys* spp. It is noted that AA1 is seemingly forked in Styloperlidae (Fig. 6 in [25]; unpubl. data). However, given that AA1 is simple in all other Plecoptera, we assume that a fusion of the anterior-most branch of AA2 with AA1 occurs in the Styloperlidae. This interpretation implies an AA2 with more than 3 branches in the family.

Another useful character state is ‘in fore- and hind wing, M occasionally with more than two branches’ (as opposed to ‘with two branches only’; orange on Fig. 8d). Among Plecoptera, this state occurs only in *carpenteri* and *Pteronarcys* spp. (pers. obs. as for the hind wing). Affinities of the new species with *Pteronarcys* spp. are further confirmed by its large size. According to Stewart & Stark (2008; [26]), among the Pteronarcyidae, a body length above 23 mm is diagnostic of the genus *Pteronarcys* (less than 20 mm in species of the genus *Pteronarcella*). The species *sinitshenkovae* **sp. nov.**, with a body length of 25 mm, is then likely to be closely related to this genus (although body size is perhaps not an ideal diagnostic character state). Also, both *sinitshenkovae* **sp. nov.** and *Pteronarcys* spp. have an area between RA and RP in the forewing with a constriction opposite the ra-rp cross-vein, a trait absent in *Pteronarcella badia* (Fig. 1a, b, e, f).

Finally *sinitshenkovae* **sp. nov.** lacks a character state shared by all *Pteronarcys* spp., namely ‘in forewing, area between R/RP and M basal to the fork of M broad, with cross-veins’ (as opposed to ‘without cross-veins’; orange on Fig. 8e; cross-veins present in Diamphipnoidae and some other large-sized Antarctoperlaria). It can then be excluded from this genus. The proposed new genus (and species) is therefore supported.

We have examined the relevance of additional characters. The occurrence of cross-veins between branches of AA2 has been indicated as diagnostic of the Pteronarcyidae [26]. However this state is not present in *sinitshenkovae* **sp. nov.** (Fig. 8f). Given the number of character states supporting the close affinities of this species with *Pteronarcys* spp., we assume that the occurrence of cross-veins in the anal area is homoplasic within Pteronarcyidae.

Regarding characters of external morphology other than wing venation, Stewart & Harper (1996; [27]) list the occurrence of three ocelli as diagnostic of the Pteronarcyidae (this character is later abandoned [26]). Three ocelli are present in *sinitshenkovae* **sp. nov.** but its phylogenetic relevance is unclear as this condition is widespread among Plecoptera.

We have also attempted to interpret the male terminalia of *sinitshenkovae* **sp. nov.** based on the very well-preserved specimen CNU-PLE-NN-2015001 (Fig. 2d,d’) and on data on terminalia of extant species [14]. We need to emphasize here that the interpretation of terminalia in fossil insect imprints is made difficult by the overlap of elements initially located at different levels. Inconclusive attempts have been made on material from the same locality [28].

**Fig. 9:**
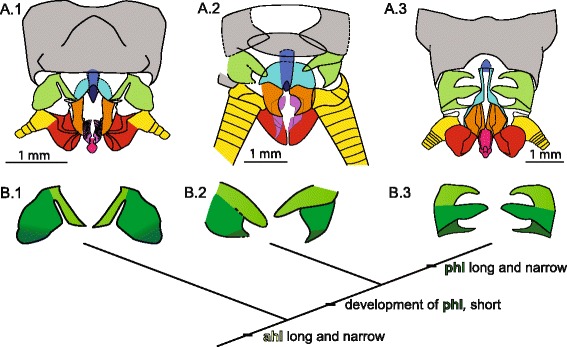
Comparation of posterior part of male postabdomen of three species (including *Pteroliriope sinitshenkovae* tax. et sp. nov.) belonging to all known genera of the family Pteronarcyidae (**a**), with same color coding for the same segments (9^th^ segment, gray; hemitergal lobe, green; ahl, light green; mhl, moderate green; phl, dark green; ip, dark blue; lb, light blue; pgp, orange; sbl, red; abs, purple; sa, pink; cerci, yellow), and the hypothesis of evolution of segments of the hemitergal lobes (**b**). **a.1**, **b.1**, *Pteronarcella badia* (Hagen, 1874) [12], modified from Fig. 35 in [14]; **a.2**, **b.2**, *Pteroliriope sinitshenkovae* tax. et sp. nov.; **a.3**, **b.3**, *Pteronarcys scotti* Ricker, 1952 [29], modified from Fig. 64 in Nelson and Hanson, 1971

We propose to compare the fossil species (Fig. 9a.2) with *badia* (Fig. 9a.1) and *Pteronarcys scotti* Ricker, 1952 ([29]) (Fig. 9a.3). The various elements we identified in *sinitshenkovae* **sp. nov.** were more easily matched with those recognized in *badia*. For example, both *sinitshenkovae* **sp. nov.** and *badia* possess well-developed lateral braces (lb; turquoise on Fig. 9a) and pointed paragenital plates (pgp; orange). We focused on the lobes of the hemitergites of the tenth segment (composed of ahl, mhl, and phl; green on Fig. 9a), because they exhibit an elaborate shape. In *badia* the hemitergites of the tenth tergum are mediodorsally divided into two lobes (Fig. 9a.1). In *Pteronarcys scotti* three lobes can be identified (Fig. 9a.3). In *sinitshenkovae* **sp. nov.** three lobes can be identified (Fig. 9a.2), but their shapes differ strongly from those in *Pteronarcys scotti*. We considered a number of hypotheses of topographic homology and selected the most parsimonious one (i.e., the one implying the smallest amount of transformations). To assist comparison we opted for the following color-coding: ahl, light green; mhl, middle green; phl, dark green; a lobe herein assumed to encompass both mhl and phl (undifferentiated) is indicated by shaded limits (Fig. 9b).

In order to explain the occurrence of two lobes in *badia* and three in *Pteronarcys scotti*, one possibility is that the posterior-most lobe in the former divides into two (which then are mhl and phl). Under this scenario (Fig. 9b) *sinitshenkovae* **sp. nov.** can be interpreted as exhibiting an intermediate condition, with the pointed, posterior process being a weakly individualized phl. In summary the development of phl would compose an apomorphy shared by *sinitshenkovae* **sp. nov.** and *Pteronarcys* spp. This hypothesis suggests that a long and narrow ahl is a plesiomorphy (it is present in *badia*, *sinitshenkovae* **sp. nov.**, and most species of *Pteronarcys*). This investigation supports the view that *Pteroliriope sinitshenkovae* **tax. et sp. nov.** is more closely related to *Pteronarcys* spp. than to species of the genus *Pteronarcella*, a hypothesis already supported by characters evaluated above.

### Evolutionary implications

Most contributions on the systematics of fossil stoneflies make extensive use of paraphyletic taxa. This approach is an impediment for current approaches focusing on divergence date estimates, because the support for the placement of particular fossil species cannot be easily evaluated. Also, the positive identification of fossil Antarctoperlaria is hindered by the lack of data on putative derived character states in the wing venation. Yet the fossil record indicates that by the Jurassic, crown-Plecoptera had already experienced a sequence of divergence events. Among the Euholognatha, both lineages *Leuctrida* and *Capniida* were already present [5, 6, 30] (the earliest known *Leuctrida*, *culonga* Sinitshenkova, 2011 [31], is Triassic). The discovery of *Pteroliriope nec Pteronarcys sinitshenkovae* complements this approach for the Systellognatha, for which divergence dates have been difficult to estimate. It demonstrates that the Pteronarcyoidea and the Perloidea (sensu Zwick, 2000 [32]) had already diverged ca. 165 million years ago, and that divergence events within the Pteronarcyoidea had already occurred.

Given that the earliest members of a given lineage are unlikely to be recovered from the fossil record, the recently proposed 170 million years divergence date estimate between the two major lineages of Plecoptera (viz. Antarctoperlaria, and Euholognatha & Systellognatha) proposed by Misof et al. (2014; [33]) clearly is an underestimate, given that *sinitshenkovae* is nested well within the Systellognatha Therefore, this deep divergence event very probably took place in the Triassic, or even in the Permian.

## Conclusions

The abundant data on *Pteroliriope sinitshenkovae* **tax. et sp. nov.** allowed us to demonstrate that this species belongs to the Pteronarcyidae, hence the group had already diverged ca. 165 mya. Our studies demonstrate the need for (re-)investigations of the systematics of fossil stoneflies to further refine divergence date estimates for plecopteran lineages. In that endeavour the positive exclusion of fossil species from particular taxa, formalized with combinations such as ‘*Pteroliriope nec Pteronarcys sinitshenkovae*’, will be a useful methodological improvement.

## Methods

### Wing venation homologies, and abbreviations

We follow the serial insect wing venation groundplan [34, 35]), and homologies proposed by Béthoux (2005, [21]) regarding Plecoptera wing venation. The corresponding wing venation nomenclature is repeated for convenience: ScP, posterior Subcosta; RA, anterior Radius; RP, posterior Radius; M, Media; MA, anterior Media; MP, posterior Media; Cu, Cubitus; CuA, anterior Cubitus; CuP, posterior Cubitus; AA: anterior Analis; AA1: first anterior Analis; AA2, second anterior Analis. The strong and oblique cross-vein occurring between M and CuA near the wing base is referred to as the ‘arculus’. Stoneflies commonly possess additional specialized cross-veins. They are usually referred to according to the veins they connect. For example, the rp-ma cross-vein connects RP and MA (and see [6]). For figures, right and left forewings are indicated as RFW and LFW respectively, and right and left hind wings as RHW and LHW, respectively. Folds are indicated as ‘f’ when necessary.

Terminologies and abbreviations for postabdominal elements follow a previous contribution [14]: ahl, anterior hemitergal lobe; mhl, median hemitergal lobe; mhp, mesal hemitergal lobe; phl, posterior hemitergal lobe; ip, inner part of supra-anal process; lb, lateral brace; pgp, paragenital plate; sbl, sub-anal lobe; abs, apical region of dorsal section of supra-anal process; sa, supra-anal process; vp, vaginal projections.

### Fossil material

The fossil material is deposited in the Capital Normal University (CNU; Dong Ren, Curator), was examined using a Leica MZ12.5 or a Zeiss SteREO Discovery V8 dissecting microscope and illustrated with the aid of a drawing tube, under dry and ethanol conditions. The resulting draft drawings were complemented during the inking process (itself performed using Adobe Illustrator CC).

### Extant material

Specimens belonging to *Pteronarcella badia* (Hagen, 1874) were prepared to complement available data on the wing venation of this species. Specimens were collected (11.vi.2010, Colorado) and determined by BK. Wings were cut off and mounted in white Euparal medium (Asco Laboratories, Manchester, UK). Specimens are deposited at the Entomology Laboratory of Muséum National d’Histoire Naturelle, Paris (France). Specimen numbers are as follows: MNHN.EP654 – MNHN.EP662.

Cladotypes and paracladotypes (belonging to *californica* Newport, 1851 and *princeps* Banks, 1907) were collected and determined by BK (2.vi.2010, Colorado; 23.v.2007, California; respectively). Specimens are deposited at the Entomology Laboratory of Muséum National d’Histoire Naturelle, Paris (France).

### Photographs

Photographs were taken using a digital camera Canon EOS 550D or EOS 5D Mark III, coupled to a Canon 50 mm macro lens, or to a MP-E 65 mm macro lens, both equipped with polarizing filters. Resulting photographs were optimized using Adobe Photoshop CS6. Unless specified, reproduced photographs of fossil specimens are dry-ethanol composites.

### Nomenclature

We use both the traditional, Linnaean nomenclatural procedure, governed by the International Code of Zoological Nomenclature [36]), and the cladotypic one ([6, 23, 37, 38] for taxa; [39, 40] for species). The traditional approach was used to warrant the validity of the newly described species. The cladotypic approach was used for its assumed higher optimality [23].

### Character states distribution

We refrained from engaging in a formal, cladistics analysis because important data on several fossil and extant Plecoptera remain to be published. Yet, in order to assess the systematic affinities of the new species, we mapped morphological character states onto a phylogenetic backbone (Fig. 8). The phylogenetic backbone is derived from a former analysis [32] complemented by data from further publications [1, 41]. Morphological data was obtained from several contributions (mainly [1, 3, 13, 14, 21, 25–27]). As for Diamphipnoidae, a very peculiar family of Antarctoperlaria, we relied on Illies (1960; [42]) and on new, unpublished data.
